# Identifying Topics for E-Cigarette User-Generated Contents: A Case Study From Multiple Social Media Platforms

**DOI:** 10.2196/jmir.5780

**Published:** 2017-01-20

**Authors:** Yongcheng Zhan, Ruoran Liu, Qiudan Li, Scott James Leischow, Daniel Dajun Zeng

**Affiliations:** ^1^ Department of Management Information Systems Eller College of Management The University of Arizona Tucson, AZ United States; ^2^ The State Key Laboratory of Management and Control for Complex Systems Institute of Automation Chinese Academy of Sciences Beijing China; ^3^ University of Chinese Academy of Sciences Beijing China; ^4^ Mayo Clinic Scottsdale, AZ United States

**Keywords:** electronic cigarettes, topic modeling, Latent Dirichlet Allocation, social media, infodemiology

## Abstract

**Background:**

Electronic cigarette (e-cigarette) is an emerging product with a rapid-growth market in recent years. Social media has become an important platform for information seeking and sharing. We aim to mine hidden topics from e-cigarette datasets collected from different social media platforms.

**Objective:**

This paper aims to gain a systematic understanding of the characteristics of various types of social media, which will provide deep insights into how consumers and policy makers effectively use social media to track e-cigarette-related content and adjust their decisions and policies.

**Methods:**

We collected data from Reddit (27,638 e-cigarette flavor-related posts from January 1, 2011, to June 30, 2015), JuiceDB (14,433 e-juice reviews from June 26, 2013 to November 12, 2015), and Twitter (13,356 “e-cig ban”-related tweets from January, 1, 2010 to June 30, 2015). Latent Dirichlet Allocation, a generative model for topic modeling, was used to analyze the topics from these data.

**Results:**

We found four types of topics across the platforms: (1) promotions, (2) flavor discussions, (3) experience sharing, and (4) regulation debates. Promotions included sales from vendors to users, as well as trades among users. A total of 10.72% (2,962/27,638) of the posts from Reddit were related to trading. Promotion links were found between social media platforms. Most of the links (87.30%) in JuiceDB were related to Reddit posts. JuiceDB and Reddit identified consistent flavor categories. E-cigarette vaping methods and features such as steeping, throat hit, and vapor production were broadly discussed both on Reddit and on JuiceDB. Reddit provided space for policy discussions and majority of the posts (60.7%) holding a negative attitude toward regulations, whereas Twitter was used to launch campaigns using certain hashtags. Our findings are based on data across different platforms. The topic distribution between Reddit and JuiceDB was significantly different (*P*<.001), which indicated that the user discussions focused on different perspectives across the platforms.

**Conclusions:**

This study examined Reddit, JuiceDB, and Twitter as social media data sources for e-cigarette research. These mined findings could be further used by other researchers and policy makers. By utilizing the automatic topic-modeling method, the proposed unified feedback model could be a useful tool for policy makers to comprehensively consider how to collect valuable feedback from social media.

## Introduction

Electronic cigarettes (e-cigarettes) have become increasingly popular in recent years. As a new type of nicotine delivery system, e-cigarettes, as defined by the US Food and Drug Administration (FDA), are battery-operated products designed to deliver nicotine, flavor, and other chemicals in aerosol form [1]. Although the FDA has expressed concern about e-cigarettes because they are not fully studied, the market has experienced tremendous growth. The sales of e-cigarette products were £3.9 billion globally, and £1.7 billion in the US, according to data from Euromonitor International [2]. The growth rate was estimated to be 24.2% per year through 2018 [3]. The fast market development has led to ongoing discussions and debates about the use of e-cigarettes, prompting significant research interests and policy concerns [4-6].

Many e-cigarette studies have used the survey method to collect information on the pattern of usage [7-16]. The survey sample was usually the general population [8,11,13-16] or current or former smokers [7,9,10,12]. The survey method included Internet survey [7,9,10,11,13,14,16], telephone survey [8], mail-in survey [15], and interview [12]. Some surveys only drew samples from one country, such as the United States [10,15,16], United Kingdom [7,9], and the Czech Republic [12], but others used international samples [8,11,13,14]. The survey questions included e-cigarette awareness, use, harm and benefit perception, and preferences. Other demographic information and smoking status were collected as well. The survey method provided evidence to lay a solid scientific foundation for public health legislation. However, surveys are usually time and money consuming. Social media, as a new channel to access to user-generated content, provides opportunities to collect large volumes of data conveniently.

The rapid growth of online communities and social media provides a new approach in collecting evidence for policy-making processes. Large social media platforms, including Facebook, Twitter, YouTube, and Reddit, enable new channels for e-cigarette users to share information and experiences. These platforms have provided efficient methods of information access for health surveillance and social intelligence [17,18]. E-cigarettes, as an emerging substitute for combustible cigarettes, are broadly studied from the perspective of social media as well. Vapor shop owners rely heavily on social media or other online communities to promote e-cigarette products by offering price discounts, specials, and loyalty programs [19].

More insights were generated from studies based on specific social media platforms. For example, one study found that the vast majority of e-cigarette information on YouTube promoted their use and depicted it as socially acceptable [20]. Another study discovered that e-cigarette-related videos usually highlighted e-cigarettes’ economic and social benefits [21]. Hua and colleagues [22] studied YouTube videos and found e-cigarette users’ puff duration was approximately twice as long as puff duration for conventional smokers. Twitter also appeared to be an important marketing platform for e-cigarettes [23]. Marketing strategies and locations of use were studied and identified from e-cigarette-related tweets [24]. Cole-Lewis and colleagues [25] conducted a thorough content analysis of e-cigarette-associated tweets and identified possible trends of e-cigarette usage growth. Topic modeling was used to examine tobacco-related tweets [26]. A supervised machine learning technique was used on Twitter data to predict the themes of posts, with fairly sound accuracy [27].

E-cigarettes are also discussed on forums. Reddit, one of the most comprehensive forums on the Internet, was used as a source to identify vulnerable populations [28] and e-liquid categories [29]. In addition to Reddit, data from three e-cigarette forums, Electronic Cigarette Forum, Vapers Forum, and Vaper Talk, were used to analyze e-cigarette-related symptoms [30]. Chen and colleagues [31] extracted contextual factors and conducted topic-modeling techniques on data from Reddit and other forums to study e-cigarette and hookah use. Social media platforms are often linked; thus, combined analyses of social media is interesting. Recently, a research paper examined the marketing strategies of leading e-cigarette brands on multiple social networking sites including Twitter, Facebook, Google+, and Instagram, providing a first step in understanding multiple social networking site marketing [32]. Their findings showed that studying the user-generated content from multiple social media platforms could be of great importance to understand the e-cigarette market’s status quo.

Moreover, we have noticed that different social media platforms have different characteristics, both for posts and users. For instance, Reddit is essentially an online bulletin system that includes all kinds of discussions [33]. As one of the most popular forums in the world, Reddit has comprehensive content about e-cigarette topics, including policy discussions, experience sharing, and promotions. Twitter, on the other hand, is efficient at information transmission. Using the retweeting mechanism, information spreads quickly through the network. In comparison, JuiceDB is a relatively new platform focusing only on e-juice product reviews [34]. The contents are limited to flavor discussions. Studying e-cigarette topics on different platforms and conducting cross-platform analysis would be of great significance because it will provide insights into how consumers and policy makers can make good use of social media to track e-cigarette-related content and adjust their decisions and policies. New e-cigarette research angles could also be generated with the help of technical tools from information science. In this research, we are interested in automatically identifying topics behind massive posts, which could be used to provide real-time support to policy makers. Furthermore, our paper aims at exploring the possibility of combining the results from multiple platforms. We provide valuable insights from the data and propose an automatic approach to generate these insights.

## Methods

### Data Collection and Preprocessing

In a previous study, we collected data from Reddit [29]. A total of 34,051 e-cigarette flavor-related posts were collected from Reddit from January 1, 2011, to June 30, 2015. In practice, there was some noise in the posts due to semantic ambiguity. We considered words not related to e-cigarettes as noise and eliminated posts that only contained noise keywords. For instance, the Apple watch is an electronic product produced by Apple Inc. Thus, the posts only containing the keyword “Apple watch” should not be covered in our analysis. Finally, a total of 27,638 unique e-cigarette flavor-related posts were identified for analysis.

Data from JuiceDB were collected by using its public application program interface (API). We collected 14,433 JuiceDB e-liquid reviews from June 26, 2013 to November 12, 2015. The dataset was comprised of reviews on e-liquids including overall rating, subrating of e-liquid components, and detailed comments.

We also collected some data from Twitter. We created crawling agents and simulated human behavior in the searching page of Twitter to retrieve historical data from January 1, 2010 to June 30, 2015. We used the keywords “e cigarettes,” “electronic cigarettes,” “ecigarettes,” “ecigs,” “smoking electronic cigarettes,” “smoking ecigarettes,” and “smoking ecigs” in the searches and collected 353,984 tweets. Compared with Reddit, Twitter is good at information transmission, which makes it an important platform for advertising and social media campaigns. Results from the Reddit dataset showed that the e-cigarette ban debate was an interesting discussion topic. “E-cig ban” and “e-cigarette ban” were general keywords describing the topic. Thus, we used these keywords to collect data and analyze the detailed discussion topic on Twitter. Some tweets were not written in English. They were collected because they used English hashtags that contained the keywords. In order to analyze English tweets only, we filtered out other tweets by using a stop words list to detect the most probable language the tweet was written in. Finally, we collected 13,356 tweets that were valid for analysis.

### Data Analysis

We used natural language processing (NLP) and Latent Dirichlet Allocation (LDA), which are information science techniques, to analyze the data. “Natural language” means the language used by humans, whereas processing means using computers to understand natural language input [35]. By enabling the use of automated methods that represent the relevant information in the text with high validity and reliability, NLP facilitates tasks such as information retrieval, analysis, and prediction in health areas [36]. Because it is difficult and time consuming for users to manually handle the huge amounts of reviews or posted data, we needed to use NLP techniques to help the computers understand the meaning of human languages. Specifically, we used basic NLP methods, including tokenization, stop words, and stemming, to process the contents of reviews and posts with the help of the Python Natural Language Toolkit (NLTK) package [35].

LDA is a generative model for unsupervised topic modeling that automatically discovers hidden topics from a set of documents, such as posts, reviews, or tweets in this study, each of which contains a bag of words [37]. The algorithm generates a given number of topics for a specific set of documents. Each document is considered to be a mixture of several topics, and a topic is characterized as a distribution of words [37]. By understanding the topic distributions among documents and the word distributions among topics, hidden information in the text could be found automatically. We used the Python package gensim to conduct LDA analysis [38]. The data processing steps are shown in Multimedia Appendix 1. Multimedia Appendix 2 shows the details of our LDA-based e-cigarette topic analysis model.

Practically, it was challenging to determine the number of topics in the LDA method. We used the hierarchical Dirichlet process (HDP-LDA) to evaluate our decision, which was also supported by the Python gensim package [39]. In the HDP-LDA model, the number of topics could be unbounded and learned from the data. We estimated the probability weight associated with each topic using the Reddit dataset. Finally, we decided to use five topics in the analysis.

The output of LDA in this study was a set of topics and the main words associated with each topic. For example, 13,356 tweets were treated as the input after preprocessing by the NLP tools. After LDA processing, five topics with associated words were summarized from these tweets. Consider each of the topics as a group. Every post belonged to one of the groups based on the words it contained.

## Results

### Dataset Analyses

We performed LDA on the three datasets. The number of topics for each dataset was set to five. For a specific topic, the top 20 associated keywords are listed in Table 1.

**Table 1:** Top five topics and keywords for posts from Reddit, JuiceDB, and Twitter.

Platform and topic		Keywords^a^
**Reddit**		
	1. Individual trades and vendor promotions	Liquid, size, mini, sold, brand, shipping, free, cream, retail, price, sample, purchase, list, prices, items, high, left, love, prefer, natural
	2. Flavor-related experiences and sentiments	Juice, flavor, good, flavors, vape, taste, great, juices, well, sweet, liquid, tastes, menthol, love, tank, nice, pretty, coffee, hit, find
	3. E-liquid components	Strawberry, flavor, VG, juice, vanilla, cream, custard, thanks, vapor, banana, PG, flavors, TFA, apple, mL, milk, 12 mg, bottles, menthol, 30 mL
	4. Relationship with traditional tobacco products	Tobacco, nicotine, vaping, smoking, cigarette, people, smoke, ecig, quit, products, health, product, year, electronic, know, companies, pack, stop, addiction, quit
	5. Personal experiences and questions	Time, know, well, feel, best, love, long, pretty, thought, start, find, want, favorite, give, question, experience, idea, hear, start, thanks
**JuiceDB**		
	1. Throat hit and vapor production	Throat hit, VG, vape, coil, tank, cloud, use, RDA, PG, vapor, max VG, liquid, dripper, high, drip, vapor production, price, higher, 50/50, 6 mg
	2. Fruit and cream flavors	Sweet, like, strawberry, exhale, flavor, nice, get, really, fruit, fruity, vape, cream, inhale, taste, candy, good, tart, well, menthol, little
	3. Cream, tobacco, and seasonings flavors	Sweet, like, creamy, rich, exhale, custard, cinnamon, get, tobacco, nice, vanilla, inhale, good, banana, cream, really, caramel, vape, smooth, hint
	4. Product promotion and recommendation	Try, vape, bottle, great, juice, order, favorite, recommend, best, flavor, day, love, time, first, adv, go, would, price, amaze, definite
	5. Vaping experiences	Like, steep, try, taste, really, get, good, vape, would, bottle, don’t, much, first, got, smell, think, bit, better, still, even
**Twitter**		
	1. Euecigban	Euecigban, eu, save, tobacco, stop, smoke, live, vaper, help, swof, try, want, people, million, smoker, please, go, via, need, product
	2. New York and noecigban	Vape, smoke, Twitter, come, pic, health, public, nyc, euecigban, cig, ad, noecigban, like, via, citi, call, propose, look, tobacco, news
	3. General discussion of e-cigarette ban	Vape, smoke, vote, blog, post, huge, electroniccigarette, consequence, citi, include, council, new, school, report, fda, house, county, harm, propose, cig
	4. Petition	Sign, vape, health, flavor, RT, want, tobacco, petitition, euecigban, say, please, support, sale, regulate, us, minor, use, propose, govern, plane
	5. Noecigban and freevape	Vape, public, noecigban, vaping, sale, smoke, place, bill, minor, freevape, new, indoor, use, would, cig, call, consider, New York, lawmaker, wale

^a^ PG: propylene glycol; RDA: rebuildable dripping atomizer; RT: retweet; TFA: the flavor apprentice; VG: vegetable glycerin.

#### Reddit Dataset Analysis

The first topic was about purchasing e-cigarette products. It contained vendor promotions and advertisements, but also individual trading information. The keywords included product descriptions and prices. Topic 2 was flavor-related experiences and sentiments. People discussed their vaping experience with specific flavors and expressed their sentiment or evaluation. Topic 3 was the discussion of e-liquid components. It is known that e-liquid consists of vegetable glycerin (VG), propylene glycol (PG), nicotine, and flavors [40], most of which showed up in this topic. Topic 4 was about the relationship between e-cigarettes and traditional tobacco products. E-cigarettes were promoted as a substitute product for traditional cigarettes. Some smokers were seeking a comparison of e-cigarettes and traditional cigarettes to decide whether to switch from smoking to vaping. From the keywords, we knew that people were concerned about nicotine and addiction problems. The final topic was about personal experience and questions. The keywords included some verbs that describe the behavior of using e-cigarettes, such as “start,” “find,” or “want.”

#### JuiceDB Dataset Analysis

The outcome of LDA on JuiceDB reviews was quite different. JuiceDB is a specific platform only for e-liquid reviews and the LDA results supported this. The top five topics were narrower and more focused on e-liquids (Table 1).

Topic 1 referred to throat hit and vapor production, which were two major features of the e-cigarette vaping experience. Topics 2 and 3 were discussions of specific flavors. From the previous study, we knew that fruit and cream flavors were the most popular, which was supported by the result that these two flavors made up one topic and other flavors were a separate topic [29]. Topic 4 was related to product promotion and recommendation. Reviews could be written for different purposes, such as individual experience sharing or advertorial promotion. The last topic was vaping experience, the same as the last topic from the Reddit results.

#### Twitter Dataset Analysis

The LDA performance on the Twitter data was even more specific because we focused on the tweets related to e-cigarette bans. Almost all tweets had a URL link that brought noise to the LDA analysis. Thus, we built the LDA model after removing URL links.

Twitter is famous for its hashtag system. The hashtag is a word coming after a hash (#) sign. It is used as a label to tag the tweet to a specific group so that users can easily find and share information in a specific community. Some of the keywords (Table 1), such as “euecigban,” “noecigban,” “electroniccigarette,” and “freevape,” were actually hashtags, and they were especially designed for social media campaigns. We observed that the topics from the LDA results were quite similar to one another. Some of the keywords, such as “euecigban,” “noecigban,” and “New York,” were present in several topics. However, topics still had their own characteristics. Topics 1, 2, and 5 were related to campaigns debating e-cigarette ban regulations. Topic 3 was a general discussion of e-cigarette bans. It had “school,” “house,” and “FDA” as keywords. Topic 4 was about petitions of the social media campaign. We saw the words “petition,” “support,” “sign,” and “us” as the typical keywords. The word “RT” represents “retweet,” which indicates the fast information transmission in the petition.

### Comprehensive Analysis Across Platforms

The preceding results described different topics for different social media platforms. Generally speaking, Reddit is a comprehensive forum so the topics are more general and broader compared to JuiceDB, which is a specific platform for e-liquid reviews. The data from Twitter showed that this social media was used as a platform for campaigns. We summarize the topics in these three platforms and present our insights for policy makers. In total, there were four types of topics: promotions, flavor discussions, experience sharing, and regulation debates.

#### Promotions

Promotion as a topic included trading among e-cigarette users and sales from vendors to users. For instance, on Reddit, one example of a vendor promotion to users was:

Wednesday Purple Drank, Banana Berry Milkshake, AND Hot Cider Donut Giveaway! Coupon code inside for 15% off ALL liquids! | Vapor Trails NW.

JuiceDB had promotions as well. However, the vendor promotions on JuiceDB were written in the format of user reviews because JuiceDB did not accept advertisements. For example:

Mountain Dew-inspired flavor. I have been using this juice for a few days now and it’s actually really good! Tastes pretty close to the real Mountain Dew flavor. It’s not exactly the same flavor as the drink but it is VERY close. I recommend it!

Trading among users was another important type of e-cigarette promotion. It was common to see these posts on Reddit because the titles usually started with want to trade (WTT), want to sell (WTS), and want to buy (WTB). For example:

WTT/WTS: Avid and MBV Juice, Also a Kanger Aerotank + full 5 pack of coils.

Among all the posts, 1636 posts had WTS in their title, 895 posts were labeled as WTT, and 431 posts were WTB posts.

Reddit, as a comprehensive platform, provides a promotion platform for both vendors and individual users. Of 27,638 posts, 2962 (10.72%) are related to trading, which indicates that there exists some secondhand e-cigarette transaction channels, raising new challenges for regulation and surveillance. Teenagers, for example, could acquire e-cigarette products easily from such channels, which decreases the effectiveness of the FDA’s proposed e-cigarette ban policy. The existence of secondhand markets introduces other possible problems as well. Without regulations and standards, the product safety is not guaranteed, raising potential risks for users. More than half of the trading posts were on the supply side, which indicates that e-cigarette users tend to be capricious about preference. This phenomenon provides evidence for the necessity of further investigation.

Reddit and JuiceDB both provided detailed descriptions of e-cigarette products. Moreover, some posts linked these two platforms together. For instance, the posts in Multimedia Appendix 3 showed the close connection between the platforms.

It is possible that users might refer to several platforms to find useful information and suggestions for vaping. We examined several other platforms, including Facebook, Twitter, the Vaping Forum, UK Vapers, E-cigarette Forum, and Aussievapers. The results are shown in Table 2.

**Table 2:** Platform links.

Link	Reddit (n=27,638), n (%)		JuiceDB (n=14,434), n (%)
	Title	Content	Content
Facebook	32 (0.12)	650 (2.35)	15 (0.10)
Twitter	7 (0.03)	290 (1.05)	0
JuiceDB (Reddit)	14 (0.05)	68 (0.25)	110 (0.76)
The Vaping Forum	4 (0.01)	7 (0.03)	0
UK vapers	13 (0.05)	4 (0.01)	1 (0.01)
E-cigarette forum	0	38 (0.14)	0
Aussievapers	4 (0.01)	13 (0.05)	0

Reddit is a comprehensive platform that links many other forums and social media. However, JuiceDB seemed to be exclusively related to Reddit.

#### Flavor Discussions

Flavor was one of the most discussed topics among e-cigarette users. Both Reddit and JuiceDB had many posts related to e-liquid flavors. In previous research, we identified eight categories of flavors: fruits, cream, tobacco, menthol, beverages, sweet, seasonings, and nuts [29]. In JuiceDB, there were nine flavor categories: sweet, fruity, rich, creamy, spiced, tobacco, cool, nutty, and coffee. The two category systems were fairly consistent, providing a good schema for future research.

From the Reddit LDA results, the topic contained several keywords related to the taste of flavors, such as strawberry, vanilla, custard, banana, apple, menthol, candy, blueberry, mango, watermelon, cinnamon, peach, caramel, lemon, chocolate, honey, cake, tea, raspberry, orange, cherry, cereal, coconut, pear, grape, cookie, peanut, mint, pineapple, and coffee. This set of flavors covered the majority of flavors found in previous research [29]. Some of them, such as caramel, cereal, and coconut, were newly discovered by the LDA results.

A study about e-cigarette flavors pointed out that new flavors would come out every now and then as the e-cigarette market develops [41]. To discover new flavors manually is expensive in both time and money. Thus, our LDA approach provided a cheap and automatic way for public health departments to complete flavor lists in real-time surveillance and trend analysis.

The findings on JuiceDB were similar. However, because JuiceDB focuses on e-liquid reviews, the topics we found were more focused. Thus, fruit and cream flavors composed a single topic, whereas other flavors made up a separate one. These two topics identified by the LDA method could help us build and complete the flavor list, as well as identify new types and trends.

#### Experience Sharing

Social media is a way for e-cigarette users to share their vaping experience with one another. People may ask and answer questions about e-cigarettes. Or they simply write down their feelings after trying a particular product. For example, a Reddit user raised a question about sweet e-juice and cavities, which is shown in Multimedia Appendix 4.

Users also shared their methods of using e-cigarettes to help others improve their vaping experience. For example, a common method is called steeping. This is a special method to process the e-liquid, especially for new products. Vapers usually believe that steeping helps to disperse chemicals and flavors throughout the juice. Steeping is simple. Just shake and store in a cool, dark place to get a well-steeped e-liquid. This is an example from JuiceDB:

Steeped this juice for 4 days, the color darkened just a bit, the flavor really came out as well.

In comparison with traditional tobacco products, e-cigarettes use e-liquid to deliver nicotine and other chemicals. Thus, the method of vaping is totally different from smoking. As far as we know, e-liquid steeping is still not well studied among the literature.

Throat hit and vapor production are two other major features of using e-cigarettes. Both JuiceDB and Reddit have thousands of posts related to them. Throat hit is the feeling of smoke hitting the back of the throat [42]. Some people like it, but some do not. Typically, there are two types of e-cigarette users. The first type is smokers who have switched or are going to switch from traditional tobacco products to e-cigarettes. They are seeking a strong throat hit and thick vapor production to acquire feelings and experiences similar to smoking, as in the following example:

This juice is basically Boba’s Bounty with Banana added in. A nice tobacco/graham cracker flavor bursting with banana but not too overwhelming, it’s just right. Great vapor production and throat hit.

The other type of users have never smoked traditional tobacco products, directly adopting vaping. Thus, they are less likely to like a strong throat hit. Their sharing and recommendations are more mild in taste. For example:

Very little throat hit in my mix (50pg/50vg 6mg) but very good vapor production.

However, both types of users are more prone to like thick vapor production. We believe that the vapor helps users’ gain a visually pleasing experience. A huge amount of vapor could produce a salient social image that is perceived and evaluated by e-cigarette users, similar to traditional cigarettes [43]. The image is studied and associated with certain attributes, such as attractiveness, sophistication, and social success, which could be a possible incentive to smoke [44]. Thus, it could also motivate e-cigarette vaping behavior. Our finding suggests that most e-cigarette users enjoy the social image of vaping.

In summary, both Reddit and JuiceDB provide users a platform to share vaping experiences. JuiceDB content is in the form of reviews and focuses more on e-liquids. Reddit, however, offers more approaches for user interactions, such as questions and answers.

#### Regulation Debates

Reddit and Twitter had topics about regulations and policy debates, but JuiceDB did not. The keywords from the LDA-identified topics included “kids,” “addiction,” “house,” “quitting,” “safe,” “cancer,” “chemicals,” “government,” “drug,” “control,” “regulation,” and “harmful.” People were discussing the effect of using e-cigarettes, especially the effects on children, and the risk of diseases from chemicals. These discussions went further and led to debates on regulations and bans.

Some Reddit users expressed concerns, whereas others appealed for not banning e-cigarettes. Examples are shown in Multimedia Appendix 5.

In general, we used the keywords “policy,” “policies,” “ban,” “bans,” “regulate,” “regulates,” “regulated,” and “regulation” to search the Reddit database, finding 872 posts. We were interested in generating a basic understanding of people’s attitudes toward e-cigarette regulations. Thus, by reading through the contents, 224 posts were considered to contain personal attitudes, which are summarized in Table 3. There were 21 proponents (9.4%), 136 opponents (60.7%), and 67 neutrals (29.9%) on e-cigarette bans. The proponents raised examples from law, research findings, and moral requirements, such as negative externality to children, to support the bans. Another interesting idea to support e-cigarette regulation was legislation benefit, indicating proper regulations could bring a better environment to the e-cigarette industry and improve the quality of e-cigarette products. However, the opponents also argued from the same fields with different evidence. The most common argument came from personal experience. Vapers argued that e-cigarettes were safer than traditional tobacco products and could save hundreds and thousands of lives. From the perspective of laws, some people said, “there is no apparent direct regulatory authority in the United States to use flavors in e-cigarettes.” Politics was another approach to battle e-cigarette regulations. Some vapers believed regulations were motivated by political pressure. Furthermore, opponents appealed for actions to down bills designed to ban e-cigarettes. Cities and states mentioned in call-for-action posts included Chicago, Berkeley, Connecticut, and Utah. The existence of so many call-to-action posts leads to the observation that Reddit serves as an important platform for vapers to organize campaigns. For instance, instructions for a mail campaign against bans are presented in Multimedia Appendix 6.

**Table 3:** Regulation debates posts on Reddit (n=224).

Post themes		n (%)
**Proponents** **(9.4%)**		
	Law	1 (0.4%)
	Research	5 (2.2%)
	Moral requirement	9 (4.0%)
	Legislation benefit	5 (2.2%)
	Tax	1 (0.4%)
**Opponents (60.7%)**		
	Personal freedom	5 (2.2%)
	Safer product	52 (23.2%)
	Law	4 (1.8%)
	Politics	8 (3.6%)
	Employee efficiency	1 (0.4%)
	Research	8 (3.6%)
	Call to action	51 (22.8%)
	How to oppose	7 (3.1%)
**Neutrals (29.9%)**		
	Possible regulation	11 (4.9%)
	Current regulation status	23 (10.3%)
	Regulation effect	15 (6.7%)
	Company rule	17 (7.6%)
	Comparison	1 (0.4%)

Correspondingly, some vapers looked for suggestions to oppose e-cigarette bans, not only federal or state regulations, but also company and university rules.

Some posts were neutral, including forecasting possible future regulations, introducing the current regulation status, analyzing regulation effects, and discussing company-specific rules. Some posts compared e-cigarettes and other addictive products, such as junk food, to discuss regulations on e-cigarette bans.

Twitter, on the other hand, focused more on information transmission. Tweets are restricted to less than 140 words, so they contain much less information than a complete Reddit post. Thus, the contents on Twitter were more straightforward and less descriptive. Twitter users tended to use other websites as references to support their point rather than describe it in detail. For instance:

RT @DeLaConcha: RT @tobacconistu: Judge rules FDA cannot ban E-Cigarettes [URL].

Twitter is also famous for its social networking function. Users connect to one another by following relationships. By retweeting posts from other users, information is quickly transmitted all over the world. Thus, the contents are more timely than Reddit posts. For example, an e-cigarette ban proposal in Coconino County could be tracked on Google as early as April 8, 2014. In our dataset, there was a tweet directing to this page right after it was published.

Finally, as we have mentioned, Twitter is a well-known platform for social media campaigns. By using certain hashtags, users become involved and influence specific topics. Ideas spread quickly through such campaigns. The hashtags #euecigban, #noecigban, and #freevape were broadly used on Twitter.

There were 3118 tweets containing the hashtag #euecigban, 916 posts containing the hashtag #noecigban, and 299 posts containing the hashtag #freevape. We analyzed the same number of posts for each hashtag group. For each hashtag, we randomly picked out 299 posts (the total number of posts that #freevape had), analyzed the content, and classified them into themes, as shown in Table 4. All the themes were against e-cigarette regulations, except for two:

1. No harm: tweets with this theme argued that e-cigarettes should not be banned because their use has little or no negative impact on human health, especially for 0 mg nicotine e-liquid.

2. Smoking cessation and saving lives: this theme stated that e-cigarettes should not be banned because e-cigarettes could act as a substitute for traditional tobacco and, therefore, e-cigarettes could help users quit smoking and save lives.

3. Pharma interests/tax income: some tweets argued that e-cigarette bans were proposed because of the interests of traditional tobacco/pharma companies or taxation from the sales of traditional tobacco.

4. Biased research: some people thought the evidence from research that supports e-cigarette bans was biased.

5. Personal freedom and rights: some people believed banning e-cigarettes was a violation of personal freedom and rights.

6. Simple opposition: some tweets just opposed e-cigarette regulations without providing any evidence.

7. Call to action: tweets in this theme were appealing for some action to oppose the ongoing bills. Usually, it was an imperative sentence with keywords “support,” “sign,” and “action.”

8. Only tag: these tweets contained a hashtag but not any other text content. Usually these tweets had URLs or pictures, which were not analyzed by this research.

9. Neutral descriptions: text content in the tweets were just descriptions without personal attitudes.

**Figure 2:**
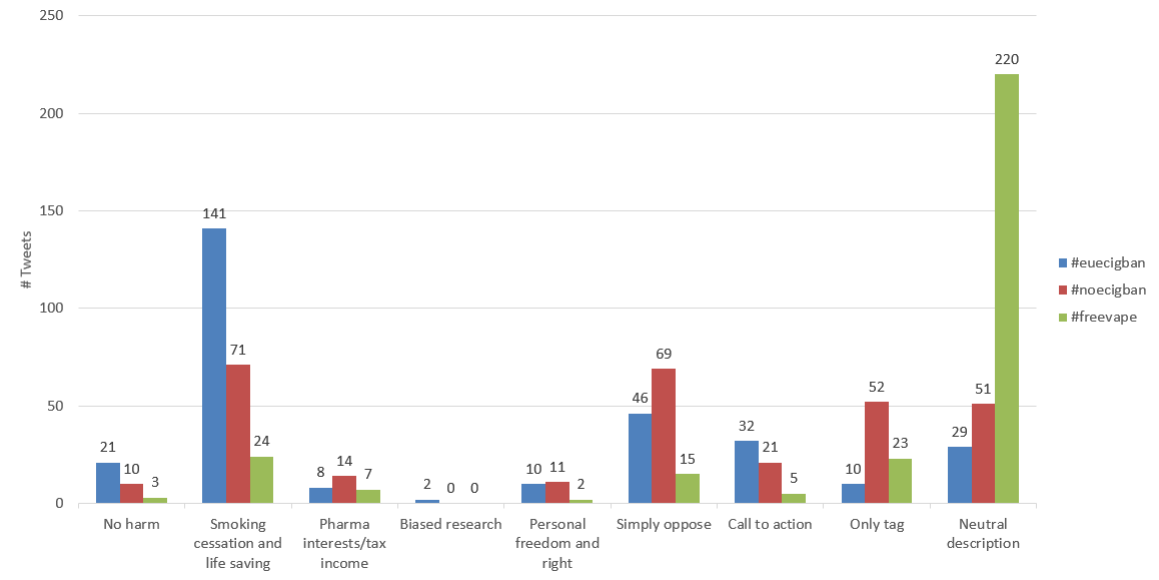
Tweet theme comparison.

Figure 2 shows the comparison of themes among these three hashtags. We observed that the #euecigban campaign was more reasonable because it had a great proportion of tweets containing evidence to support their statement. However, #noecigban focused more on direct opposition with some URLs and pictures. The campaign by #freevape seemed to be more descriptive and illustrated the current status of e-cigarettes with a neutral perspective.

In summary, Reddit, which is essentially a forum, has more user discussions and interactions than Twitter. But Twitter is good at information transmission and social media campaigns.

**Table 4:** Twitter hashtag analysis.

Hashtag and category		n (%)
**#euecigban (n=299)**		
	No harm	21 (7.0)
	Smoking cessation and life saving	141 (47.2)
	Pharma interests/tax income	8 (2.7)
	Biased research	2 (0.7)
	Personal freedom and right	10 (3.3)
	Simply opposition	46 (15.4)
	Call to action	32 (10.7)
	Only tag	10 (3.3)
	Neutral description	29 (9.7)
**#noecigban (n=299)**		
	No harm	10 (3.3)
	Smoking cessation and life saving	71 (23.7)
	Pharma interests/tax income	14 (4.7)
	Biased research	0 (0.0)
	Personal freedom and right	11 (3.7)
	Simply opposition	69 (23.1)
	Call to action	21 (7.0)
	Only tag	52 (17.4)
	Neutral description	51 (17.1)
**#freevape (n=299)**		
	No harm	3 (1.0)
	Smoking cessation and life saving	24 (8.0)
	Pharma interests/tax income	7 (2.3)
	Biased research	0 (0)
	Personal freedom and right	2 (0.7)
	Simply opposition	15 (5.0)
	Call to action	5 (1.7)
	Only tag	23 (7.7)
	Neutral description	220 (73.6)

#### Differences Across Platforms

The comprehensive analysis in the previous part presented the results summarized from all the data available. However, another interesting question came from the differences across platforms; specifically, whether the posts from different platforms had different topic distributions. As shown previously, the dataset collected from Twitter was more related to regulation debates, whereas the datasets from Reddit and JuiceDB were more comprehensive because of the keywords selected in the data collection processes. Thus, in this study, we only compared the topic distributions between Reddit and JuiceDB.

As stated in the data analysis section, the LDA algorithm identified five topics from a collection of Reddit or JuiceDB posts. In order to compare across the platforms, we manually classified those topics into three groups: promotion, flavor, and experience. Each of the posts was categorized into one of the groups. For Reddit, the number of topics in promotion, flavor, and experience were 2152, 21,752, and 3734, respectively; for JuiceDB, the number of topics in promotion, flavor, and experience were 4203, 5196, and 5034, respectively.

We ran a chi-square test to compare the differences in topic distribution between Reddit and JuiceDB. The results showed that the topic distribution was significantly different (*P*<.001), which indicated the user discussions focused on different perspectives across the platforms.

## Discussion

### A Unified Feedback Model

**Figure 3:**
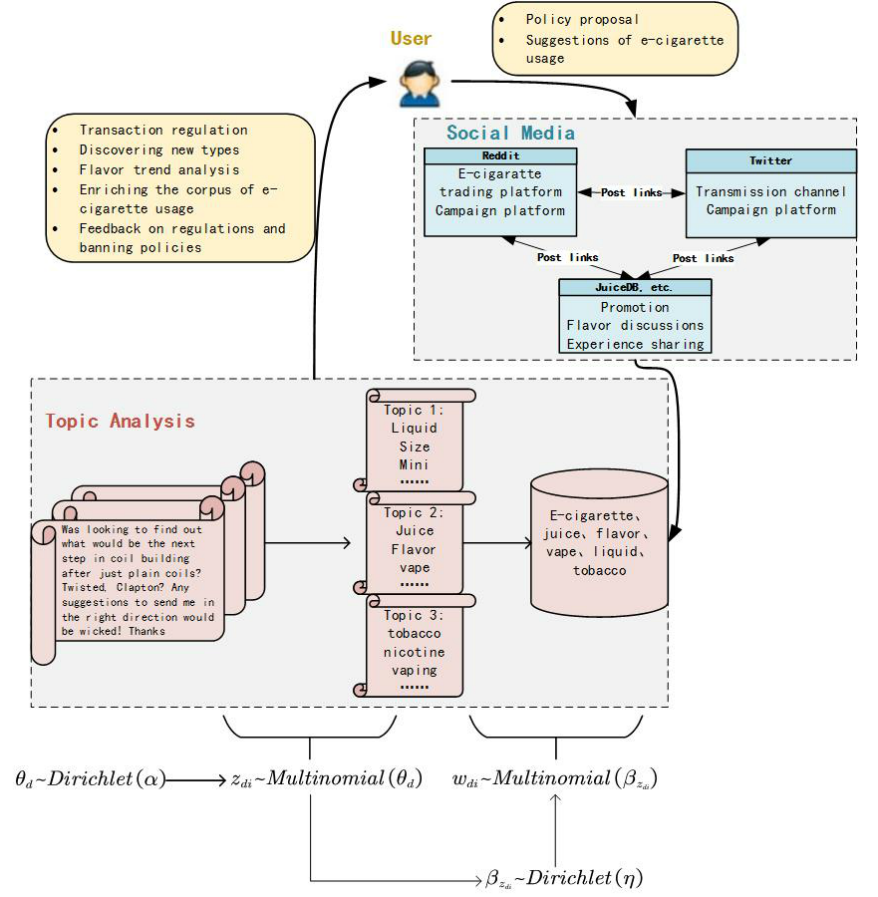
A unified e-cigarette social media feedback collection and analysis model.

We provide a general framework to analyze user-generated content from social media. After the raw materials are collected, we believe it will be much better if the topic-modeling method is used to generate some insights for further analysis. For instance, we found several topics by applying LDA methods to datasets collected from different social media. These topics are classified into four types: promotions, flavor discussions, experience sharing, and regulation debates. Compared to the results from surveys and experiments, data from social media are collected in the field and have a large data size, which provides a potential approach to generate valuable insights. Moreover, collecting data online uses less time and money than recruiting participants to complete questionnaires. Based on the previous analysis, we propose a unified model for e-cigarette policy proposals, as shown in Figure 3. In this framework, the researchers and policy makers can obtain feedback to policy proposals, which can be used as evidence to support public health policy development. Governments also have official accounts on Twitter and Facebook because they are considered as the most influential social media. Thus, policy proposals could be published by the official account on these two websites. Thanks to the high speed of information transmission, all the major social media will soon be notified. Users in different platforms will provide valuable feedback to the policy. After data collection, the topic-modeling method provides a possible approach to measure the feedback because it presents the implicit structure of the data. The topic and many other metrics can be used together to conduct public health surveillance. Although using keywords can provide a continuous record for trend analysis, the change of topics and corresponding keywords can help us identify which keywords should be listened to. As mentioned previously, topic modeling is helpful in broadening policy makers’ horizons, enriching research corpus, and detecting emerging trend.

Consider two simple examples. Assume that government departments, such as the FDA, want to collect some data about symptoms and adverse events from using different flavored e-liquids [1]. With our model, major platforms such as Twitter, Facebook, and Reddit could be considered. The topic of flavor discussions could be identified automatically using LDA methods. Posts belonging to this topic should be further examined. Furthermore, JuiceDB, serving as a second-tier platform, could provide additional information to analyze the effect of flavors. Another example is collecting public comments and thoughts for future regulations. The FDA has held three public workshops to obtain information on e-cigarettes and public health. However, our model provides another approach to collect additional information from the field. Reddit and Twitter are important platforms for regulation feedback even though they emphasize different aspects. Information transmission on Twitter is faster whereas discussions on Reddit are more detailed. Both of them provide unique angles to understand public comments. In addition, some other second-tier platforms could be useful for exploring deeper and further thoughts.

### Contributions

In summary, the rapid growth of e-cigarette user communities indicates the importance of research in this field. Social media has proven to play an indispensable role in promotions and communications. Previous research has utilized social media as the data source to study e-cigarettes. Most of them focused on only one specific platform [19-31]. Therefore, there is still a lack of comprehensive examination across multiple social media platforms. Chu and colleagues [32] used data from both Facebook and Twitter to study the marketing strategies of e-cigarette brands. This paper is inspired by the previous research, but contributes to the field by analyzing topics across the platforms using automatic topic-modeling tools. The LDA method is introduced to researchers and policy makers who are interested in data mining and machine learning. Reddit is recognized as a comprehensive forum for e-cigarette discussions, whereas JuiceDB only focuses on e-liquid reviews. Twitter has less information within each post, but is good at data transmission and campaign detection. Furthermore, the types of topics are summarized into four groups: promotions, flavor discussions, experience sharing, and regulation debates. Statistics are summarized to generate insights into the current state of e-cigarette communities. Specifically, we found (1) 11% of the Reddit posts were user trading posts, which showed evidence of the existence of a large secondhand e-cigarette trading market, raising new concerns in regulations and surveillance; (2) flavor discussions from JuiceDB and Reddit followed consistent category systems, which provided a good framework for automatically discovering new products and emerging trends; (3) experience sharing included e-cigarette vaping methods, features, and outcomes, which served as evidence of the patterns of e-cigarette use; and (4) regulation debates from Reddit could be used to collect feedback, whereas Twitter was a popular platform for a social media campaign. The topic distributions within Reddit and JuiceDB were significantly different (*P*<.001), which indicated the user discussions focused on different perspectives across the platforms. The unified feedback model we presented to collect valuable proposal feedback from social media will save policy makers’ time and money.

### Limitations

We collected data from Reddit, JuiceDB, and Twitter, which was feasible for our current research. However, several other platforms, such as Facebook and E-cigarette Forum, could be considered to expand the current dataset for further analysis. We only collected regulation-related data from Twitter, but other e-cigarette-related tweets could be of interest. A more general keyword set should be created for data collection across the platforms. Moreover, the keywords “vape,” “vapor,” and “vaping” should be included in the next step of data collection. However, we still believe the research findings from the current dataset provide valid and valuable insights.

Another limitation of this paper was the lack of demographic information. Because Reddit, JuiceDB, and Twitter do not provide reliable personal characteristics, such as age and gender, we cannot divide our dataset into several subgroups to analyze the different patterns among different age or gender groups.

Finally, this study only used LDA to identify topics among posts. There are many other data mining tools that could be applied to further explore the dataset. For instance, sentiment analysis could be conducted on the regulation-related posts. Positive, neutral, or negative sentiments are an important indicator for understanding public comments.

### Future Research

We envision three possible approaches for further study. First, the LDA model could be modified and extended for further analysis. In this paper, we applied the standard LDA techniques as the topic-modeling algorithm, and the results were feasible enough to conduct some analysis. However, given the special context of e-cigarettes, we believe that some modifications to the standard LDA model could produce better and more precise results. For instance, topic-in-set knowledge could be added to achieve supervised learning [45]. Another study modified LDA to find groups in graphs, which could be helpful in finding e-cigarette promoters in social media networks [46]. Social media analysis is famous for its big data. LDA could be applied in a distributed way to process the big data as well [47]. In summary, there are many modifications to the standard LDA model, which could be further explored by us and other researchers.

Second, major types of topics are identified, each of which is interesting and makes practical sense. Some findings and discussions could be further explored. For example, individual trading is an emerging phenomenon in the e-cigarette market, which could produce potential risks to e-cigarette regulations. Vendors’ promotions are also worth studying to find patterns. Automatic emerging e-liquid detection and symptoms collection are important as well. Studying feedback on proposed policies would generate insights for policy makers to make better decisions.

Finally, the characteristics of social media platforms should be further analyzed. For example, the problem of bots, fake accounts, and spam on Twitter is worth exploring, from both a research perspective and an application perspective. It will be challenging and meaningful if we can develop an automatic filter for more accurate analysis on Twitter. The algorithm itself and the patterns of spammers are worth studying. The connections between platforms are interesting as well. If we could identify the same account across platforms, the information flow could be easily understood, providing a valuable signal for public health surveillance.

### Conclusion

Using topic modeling techniques LDA, we identified topics among posts generated by e-cigarette users. This automatic method could be used to analyze the state of the art in the e-cigarette field. New brands, flavors, and trends could be found using our method, which is of great importance to the fast-developing e-cigarette market. We compared the results from Reddit, JuiceDB, and Twitter and discussed the similarities and differences of the platforms. We hope the characteristics analyzed by this paper can be further used by other researchers and policy makers.

## Appendix

Multimedia Appendix 1 Graphical representation of the LDA model.

Multimedia Appendix 2 LDA model.

Multimedia Appendix 3 Close connections between Reddit and JuiceDB.

Multimedia Appendix 4 Sweet e-juice and cavity.

Multimedia Appendix 5 Regulation debates from Reddit.

Multimedia Appendix 6 An instruction to against e-cigarette ban by mails.

## Abbreviations

API: application program interface
E-cigarette: electronic cigarette
FDA: Food and Drug Administration
LDA: latent Dirichlet allocation
NLP: natural language processing
PG: propylene glycol
RDA: rebuildable dripping atomizer
RT: retweet
VG: vegetable glycerin
WTB: want to buy
WTS: want to sell
WTT: want to trade
