# Supplementary figures and images for: Identifying Topics for E-Cigarette User-Generated Contents: A Case Study From Multiple Social Media Platforms
Source: J Med Internet Res. 2017 Jan 20;19(1):e24. doi: 10.2196/jmir.5780 (PMC5291865; doi:10.2196/jmir.5780)

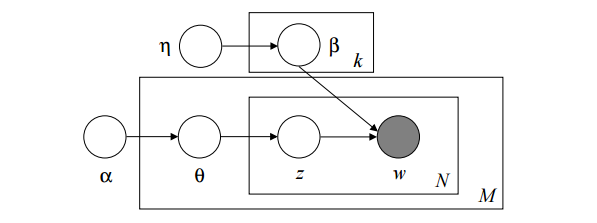

Supplement: Multimedia Appendix 1 [file jmir_v19i1e24_app1.png]
