# Supplementary material for: Identifying Topics for E-Cigarette User-Generated Contents: A Case Study From Multiple Social Media Platforms
Source: J Med Internet Res. 2017 Jan 20;19(1):e24. doi: 10.2196/jmir.5780 (PMC5291865; doi:10.2196/jmir.5780)
Supplement: Multimedia Appendix 2 [file jmir_v19i1e24_app2.pdf]

In this model, our dataset is a collection of M e-cigarette-related posts sharing the same k topics. For example, we had 34,051 posts from Reddit and they shared five topics. An e-cigarette-related post was considered to consist of several possible topics, such as regulation debates, e-liquid recipe discussion, and symptom discussion. The topics were hidden in the model; thus we did not know them beforehand. For each post, topic distribution might be different. For instance, a post might have used symptom examples to support e-cigarette regulation, which meant it was composed of a symptom discussion topic and a regulation debate topic. However, another post might have used symptoms to discuss e-liquid choice, which meant it was composed of an e-liquid recipe discussion topic and a symptom discussion topic. We denoted θ_i as the topic distribution over post i. This θ_i was different for different posts. The probability of choosing a specific topic on post i, word position j (e.g., regulation debates on the first word position of the post), was denoted as $P(z_{ij}|\theta_i)$. Topics are related to words. The regulation debates topic has a closer relationship with the words “ban,” “regulation,” and “policy,” whereas the symptom discussion topic has a closer relationship with the words “cough,” “throat hit,” and “dry mouth.” Therefore, different topics had different word distributions over the whole vocabulary. We denoted β_k as the word distribution for topic k. Therefore, a specific word w_{ij} on post i, position j had the probability For example, the word “policy” could be related both to

the regulation debate topic and the symptom discussion topic. The probability of the occurrence of this word in the post i, position j was the sum of the probability of the occurrence of this word in both of the topics in the post i, position j.

As previously mentioned, LDA is a generative model. The generative process is described as follows. First, draw topic distribution θ_i for all posts i in the collection M by $\theta_i \; Dirichlet(\alpha)$. The $Dirichlet(\alpha)$ is the Dirichlet distribution with parameter α. Then, draw word distribution for all the topics β_k in the collection of k by $\beta_k \; Dirichlet(\eta)$. Finally, for each of the word position i, j choose a topic $z_{ij} \; Multinomial(\theta_i)$, and then choose a word $w_{ij} \; Multinomial(\beta_{z_{ij}})$.

Words are the only thing we can observe from the model shown in the Multimedia Appendix 1, which is the reason why only this circle is shaded. Our goal was to identify hidden topics and topic-related keywords. By feeding the collection of posts into the model, we could estimate the parameters θ_i and β_k by using maximum likelihood estimation. Although θ_i was used to indicate topics for a given post, β_k was used to describe the meaning of topics based on the distribution of words.
