# Supplementary material for: Identifying Topics for E-Cigarette User-Generated Contents: A Case Study From Multiple Social Media Platforms
Source: J Med Internet Res. 2017 Jan 20;19(1):e24. doi: 10.2196/jmir.5780 (PMC5291865; doi:10.2196/jmir.5780)
Supplement: Multimedia Appendix 3 [file jmir_v19i1e24_app3.pdf]

Appendix 1. Close connections between Reddit and JuiceDB.

I found this vendor from JuiceDB.com (which is a good place to try out new juices with having feedback from the public before you buy so you can have an understanding of what you are getting), Called Feghali vapor. I went on their site and it's a cool black and red theme site and I noticed they sell "value" e-liquids and have a small premium line. I saw like $25 for 15 10ml bottles or $10 for 3 30ml bottles & if it’s that cheap it can’t be that good so I stuck with the premium line which already had good reviews on Juicedb. (Reddit post)

A note about their customer service: when my first order came in, the juice had a harsh quality to it. I posted on Reddit about it asking if this was normal for the juice. Within ten minutes, Kite in Cloud responded to my post and insisted on sending me a brand new bottle at no cost. They said that the nicotine must have oxidized and was responsible for the harsh throat hit. (JuiceDB review)
