# Supplementary material for: Identifying Topics for E-Cigarette User-Generated Contents: A Case Study From Multiple Social Media Platforms
Source: J Med Internet Res. 2017 Jan 20;19(1):e24. doi: 10.2196/jmir.5780 (PMC5291865; doi:10.2196/jmir.5780)
Supplement: Multimedia Appendix 4 [file jmir_v19i1e24_app4.pdf]

Appendix 2. Sweet e-juice and cavity.

Weird question here, is anyone else noticing their dental health has changed since using sweet liquids? Here's some background: I'm an avid brusher, flosser and mouthwash user. I've never had a cavity before age 30 and suddenly my visit to the dentist last week revealed that I have 5 cavities!!! I always go to the dentist (it had been 1 year since my last visit though, but I usually go once or twice a year). I've not changed my diet habits or sweets intake and I take care of my teeth. In the past year, the only difference is that I started vaping (I know I'm weird, but I've never smoked actual cigarettes). So I'm thinking that maybe the sweet juice is causing cavities. I asked my dentist, but he doesn't know a lot about vaping, but he thought maybe the sweet liquid could be a factor. I use a VP/PG mix from my local vape shop. It's usually a banana/vanilla flavor. I know this is obscure, but I wanted to see if anyone else had this problem. I can't find a lot online about it.
