# Supplementary material for: Identifying Topics for E-Cigarette User-Generated Contents: A Case Study From Multiple Social Media Platforms
Source: J Med Internet Res. 2017 Jan 20;19(1):e24. doi: 10.2196/jmir.5780 (PMC5291865; doi:10.2196/jmir.5780)
Supplement: Multimedia Appendix 5 [file jmir_v19i1e24_app5.pdf]

Appendix 3. Regulation debates from Reddit.

Post 1:

Dude, e-liquids are still cheap as fuck compared to cigarettes.... and they all have full ingredients lists on the labels anyways so if you accidentally inhaled a mixture of VG/PG, kosher paint thinner, cloud 9/relax, and cinnamon instead of a mixture of VG/PG, and flavorings only: its your damn fault/the fault of the single manufacturer that put people in danger in the first place.... we shouldn't have to risk putting start-up vaping companies out of business because not enough people bought the higher priced liquid combined with the guaranteed-to-be-high-as-hell taxes on said liquids, we shouldn't have to put up with the FDA possibly limiting the sizes of e-liquid bottles to be sold like the twats in some European countries did, and we shouldn't have to put up with the FDA possibly limiting the exact amount of flavoring to be used per mixture because "Oh noess!!! Think of the children that we refuse to actually parent!"

Post 2:

See, they can't ban flavoring itself though. It's used in so many things. From homemade candies and soda to all sorts of things. Australia has doubler's though, they sell the nicotine separately so you mix it with the flavored VG/PG. They can't regulate flavoring, VG, or PG, because those things are already so tightly regulated, it isn't feasible without disrupting so many industries.
