# Supplementary material for: Identifying Topics for E-Cigarette User-Generated Contents: A Case Study From Multiple Social Media Platforms
Source: J Med Internet Res. 2017 Jan 20;19(1):e24. doi: 10.2196/jmir.5780 (PMC5291865; doi:10.2196/jmir.5780)
Supplement: Multimedia Appendix 6 [file jmir_v19i1e24_app6.pdf]

Appendix 4. An instruction to against e-cigarette ban by mails.

1) The letters should target the Federal Minister of Health, as well as the Prime Minister. To access the mailing address of each Member of Parliament, visit http://www.parl.gc.ca/parliamentarians/en/members
Letters sent to the government do NOT need a stamp, so your letter won't cost you a thing.
2) Ask for the general authorization of electronic cigarettes
3) Focus on PERSONAL experience using electronic cigarettes
4) Give personal information on your health since picking up electronic cigarettes. If applicable, contrast your experience with conventional smoking cessations aids.
5) Give other examples. Do you have friends that made the switch? Mention it!
6) If you agree that electronic cigarettes should not be sold to minors, and that some sort of regulation is needed to make sure that e-juice is as safe as possible, let them know
7) Tell them that regulations should help electronic cigarettes be more available than regular cigarettes (Brick and Mortar shops, tasting bars, samples, etc.)
8) Tell them that regulation of electronic cigarettes should not be a barrier to the development of technical innovations, which helps provide even more options for consumers.
